# An ethogram identifies behavioural markers of attention to humans in European herring gulls (*Larus argentatus*)

**DOI:** 10.1242/bio.060016

**Published:** 2023-06-13

**Authors:** Franziska Feist, Paul Graham

**Affiliations:** School of Life Sciences, University of Sussex, Brighton, East Sussex BN1 9QG, United Kingdom

**Keywords:** Herring gull, Attentional modulation, Ethogram, Urbanisation, Human–wildlife interaction

## Abstract

Herring gulls (*Larus argentatus*) are one of few species thriving in anthropogenic landscapes. Their history of urbanisation and familiarity with people makes them a good target for studies of human–wildlife interactions. Previous research highlights a connection between food-stealing behaviour, success in anthropogenic areas, and increased attention towards humans, raising questions about the exact extent of a gull's knowledge of human food cues. To explore these, behavioural responses to human cues in a food-related context were investigated and presented in a systematic ethogram, which identified three distinct markers of attention. Head turns, approaches, and angular body position all differed significantly between control and food conditions, showing that attention towards humans in a food-related context was upregulated and reflected in behaviour. In food condition trials, head turns occurred more often and gulls faced more towards the experimenter with occasional approaches that were never seen in control conditions. Acoustic and behavioural human food-like cues alone seemed insufficient to elicit these responses, indicating that gulls specifically paid attention to the details of human behaviour or had specific knowledge of anthropogenic food items. These results show situation-dependent attentional modulation in gulls and provide a description of attentive behaviours that can be used in further study.

## Teaser

**Summary:** Urbanised herring gulls successfully benefit from anthropogenic food sources. We show that human food-centred behaviours directly modulate the attentional states of such gulls.

## INTRODUCTION

The human population is increasing, and with it comes the expansion of cities and urban landscapes (Lowry et al., 2013). Often this negatively affects local wildlife and causes species to avoid urbanised areas (Bateman and Fleming, 2012). Few manage to survive in anthropogenic landscapes, but those that do can be very successful and urban populations sometimes exceed densities found in natural environments (Bateman and Fleming, 2012).

This is true for the European herring gull (*Larus argentatus*). First instances of urban nesting occurred in the 1940s, and by the 1960s, urban breeding colonies had spread throughout the UK's coastal areas (Rock, 2005). Today, herring gulls’ overall population numbers are declining globally (BirdLife International, 2018), while urban populations continue to increase (Rock, 2005). As a result, urban herring gulls are familiar with the presence of people and anthropogenic food, which makes them an excellent target for studying the ecological and cognitive factors that contribute to the success of urbanised populations and the human–wildlife conflicts that follow.

The kleptoparasitic behaviour of herring gulls suggests that they have a high degree of behavioural flexibility (Spencer et al., 2017) and an ability to store, integrate, and use information about their environment and other individuals in decision-making (Lee and Thornton, 2021; Morand-Ferron et al., 2007), something which may have been useful for adaptation to anthropogenic landscapes (Bateman and Fleming, 2012; Plumer et al., 2014). Urban gulls have been shown to pay attention to humans and human food cues, are aware of human gaze direction (Goumas et al., 2019), and preferentially peck at a food item that has been handled by a person when given the choice between two identical food objects (Goumas et al., 2020a).

We investigated human–gull interactions in a food-related context by creating a systematic ethogram, a catalogue of behaviours exhibited by an animal that described distinct attentive behaviours, and confirmed whether urban gulls paid attention to humans in general or specifically in a food-related context.

## RESULTS

The goal of this observational study was to create a systematic ethogram to identify markers of attention toward a human in a food-related context. Our results suggest that attentive behaviours were upregulated during food condition (FC) trials but not during no-food condition (NFC) and only rarely during paper condition (PC) trials, which included simulated acoustic and behavioural food stimuli. Immature individuals exhibited significantly fewer head turns than adults, while body orientation relative to the experimenter remained unaffected by age. Group size did not significantly affect head turns, body orientation relative to the experimenter, or approach occurrence.

### The presence of a food stimulus increased head turn counts

**Fig. 1.**
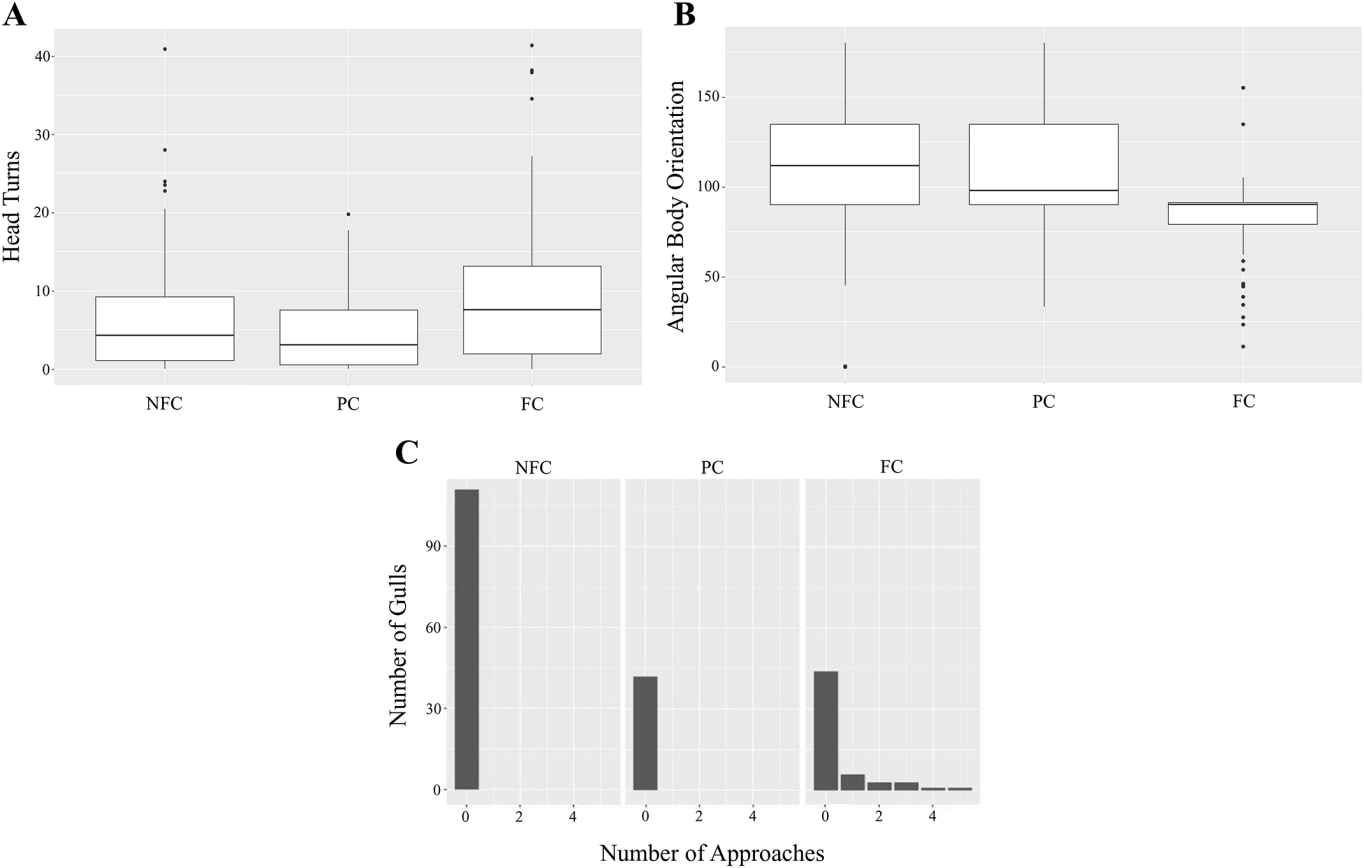
**Upregulation of attentive behaviours during food trials.** (A) The number of head turns per subject per minute during each tested condition. During FC trials, these were significantly higher than in NFC and PC trials (*P*<0.05). The horizontal bars indicate the 1st and 3rd quartiles and the median. (B) The weighted average angular body position relative to the experimenter during the three conditions, with horizontal bars highlighting the 1st and 3rd quartiles and the median. During FC trials, body orientation differed significantly from NFC trials (*P*<0.001), with more gulls facing ≤90° towards and almost none facing >115° away from the experimenter. (C) Approach instances during the three conditions. No gulls tested in NFC or PC trials approached, while 14/53 or 26.42% of gulls tested in FC trials did. Within one trial, gulls that did approach did so within one to five individual approach instances.

The first generalized linear mixed model (GLMM) showed that head turns were significantly influenced by condition and age, but not by group size (full versus null*:* x²=41.935, d.f.=6, *P*<0.001). Head turns in NFC trials differed significantly from those in FC [estimate=0.351, standard error (se)=0.156, *P*<0.05] but not from those in PC (estimate=−0.078, se=0.190, *P*=0.680) trials. Additionally, there was a significant difference between FC and PC trials (estimate=−0.429, se=0.209, *P*<0.05). This confirmed that head turns were upregulated in the presence of human food cues, suggesting increased vigilance in a food-related context (Fig. 1A). Additionally, immature individuals showed fewer head turns compared to adults (estimate=−0.304, se=0.131, *P*<0.05), indicating that vigilance, as indicated by head turns, was lower in immature individuals than in adults.

### More gulls faced towards the experimenter in the food condition

The weighted average angular body position relative to the experimenter was significantly affected by condition (Fig. 1B), but not by age or group size (full versus null: x²=41.671, d.f.=6, *P*<0.001). Body orientation differed significantly between NFC and FC (estimate=−19.054, se=5.120, *P*<0.001) but not between FC and PC (estimate=12.051, se=6.334, *P*=0.057) or NFC and PC trials (estimate=−7.003, se=5.671, *P*=0.217), with more subjects facing 90° or less towards the experimenter during FC compared to PC and NFC trials.

### Approaches only occurred when the experimenter signalled food

No trial effect on approach occurrence was found (Pearson's Chi squared test, X-squared=20.658, d.f.=15, *P*=0.148). No birds started approaching during NFC (*n*=101) or PC (*n*=40) trials, but 14 out of 53 gulls (26.42%) did approach the experimenter during FC (*n*=53) trials, with between one and five approach instances per trial (Fig. 1C). A Pearson's Chi squared test confirmed that there was an association between condition type and approach occurrence (Pearson's Chi-squared test, X-squared=41.142, d.f.=10, *P*<0.001, *n*=194). There was no significant effect of group size or age on approach occurrence.

Together, these results suggested that gulls paid increased attention to humans in possession of food (FC), which led to specific behavioural changes. Head turn counts increased significantly, more gulls oriented themselves towards or perpendicular to the experimenter, and some started approaching, which was not seen in other conditions. In contrast, the acoustic and behavioural human food-like cues given during PC trials seemed insufficient to elicit an increased number of head turns, a response only recorded in the presence of a real food item.

## DISCUSSION

We have identified three behaviours – head turns, orientation towards an experimenter, and approaches – that were significantly upregulated during FC compared to NFC trials, and only slightly in PC trials, which suggests that they are reflective of increased attention towards people in possession of food. It is hypothesised that all three behaviours allow gulls to combine attention towards a potential food source with higher vigilance and maintain possibility to escape if necessary. Since PC trials, during which human food-like behavioural and acoustic cues were mimicked, seemed insufficient to elicit similarly strong responses as those seen in FC trials, it is suggested that these attentive behaviours are specific to a foraging context and that the given cues in PC trials may have been insufficient to signal food availability or deemed irrelevant by the gulls.

### What purpose do these behaviours serve?

Approaches can be interpreted as interest in the experimenter, the food item, and, potentially, a sign of preparations for a food-stealing attempt. They only ever occurred in trials where a food item was present, which supports this idea and confirms that the stimulus presented in this condition was attractive to the gulls. The approach rate of the studied population (24.14%) was in line with what was expected based on previous reports and the experimental set-up used in this study. Previously, the approach rate of urban herring gulls towards a person in possession of food was reported to be around 26% (Goumas et al., 2019). In contrast, approaches towards a food source 8 m away from the experimenter were around 68% (Goumas et al., 2020a). In our study, the food item remained with the experimenter and so the relatively low proportion of potential food-stealers (as indicated by approach attempts) was in line with our expectations.

Head movements have previously been suggested to be a proxy for scanning behaviours (Fernández-Juricic, 2012) indicative of vigilance rates in birds (Hart and Lendrem, 1984). While foraging birds have been shown to be able to detect predation risks, raising their head for scanning increases their detection abilities (Lima and Bednekoff, 1999). Therefore, the increase in head turns can be seen as an indicator of increased vigilance towards an attractive food item. More frequent head movements may allow gulls to plan their approach accordingly, while simultaneously keeping watch for competition from conspecifics and predation risks. In this regard, the lower number of head turns seen in immature individuals may be indicative of their general lower interest in an anthropogenic foraging opportunity, a lack of boldness required to engage with humans, or avoidance of competition with adults in which they are likely to lose (Monaghan, 1980).

Lastly, subjects were observed to face 90° or less towards the experimenter if a food object was present, which could be reflective of a gull's attempt to prepare for an approach to steal food while ensuring that escape is possible in case the experimenter were to pose a threat. Predators preparing for an attack orient their head and body towards their target, which is used by prey to assess immediate predation risks (Book and Freeberg, 2015). At the same time, since humans can pose a threat to gulls (Goumas et al., 2020b), they should orient themselves in a way that allows escape if necessary, such as facing predators side on, which increases predator detection (Kaby and Lind, 2003). Losing an encounter as prey will have more serious consequences than an unsuccessful foraging attempt (van den Hout et al., 2010), which may help explain why the birds tended to favour a position that trades escape opportunities with approach conditions. The fact that the majority of birds faced away from the experimenter during NFC trials may support this hypothesis, indicating that individuals prioritise escape efficiency in situations where no foraging opportunity is available, or that they may simply have been disinterested.

### How do gulls identify food objects?

While we did show that the specific food stimulus we presented during FC trials seemed to be attractive to the gulls, this interpretation does open up questions about the mechanisms of anthropogenic food choice in gulls. Our PC control trials, which consisted of human behavioural and acoustic food-like cues without presence of an actual food item, did not provoke an upregulation in head turns or approaches and resulted in only a slight, non-significant change in body orientation. Thus, the given cues were seemingly insufficient to signal food availability, potentially owing to the fact that they were not considered relevant enough. Both this and previous studies highlight the fact that gulls specifically pay attention to humans in possession of food, and that these combined cues influence approach (Goumas et al., 2019, 2020a). Interestingly, when given the choice between two non-food items, gulls do not peck at a handled non-food item above chance levels (Goumas et al., 2020a), indicating that gulls may make a decision about the nature of an object based on the object's characteristics before attending to human cues associated with it. As such, the object itself may be used to determine whether it is indeed a food item, while human cues are used to make choices about foraging opportunities. This notion would explain why the behavioural and acoustic cues given in the PC trials were insufficient to elicit an increase in head turns similar to that seen when a real food item was presented, only leading to some changes in body orientation; initially, the gull's attention may have been captured, but the object was then identified as a non-food object, thus resulting in less attention paid to the situation. Little is known about what types of sensory cues gulls learn about and use to identify food items. It is likely that visual, olfactory, and auditory cues are used in context-dependent ways, at different distances, to identify foraging opportunities and confirm the presence of food. Despite human behaviour having been the same across PC and FC trials, there were differences in the visual and olfactory cues provided by the item, thus potentially explaining their lowered attention in a context that lacked such olfactorily and visually familiar sensory cues.

### Application of the presented ethogram

Our results indicated that urban herring gulls modified their behaviour in response to humans when food was present. Head turns, approaches, and body orientation relative to the experimenter were upregulated when gulls paid attention to a person in possession of food. As such, these behaviours can be used as markers of attention towards human cues, which will, in turn, be useful in future studies of herring gull behaviour and attention. It has been suggested that gulls’ success in urban environments may be due to their cognitive capabilities and high behavioural flexibility (Bateman and Fleming, 2012; Plumer et al., 2014), and adaptive modulation of attention may play a role in this. One important, unanswered question is how food-dependent modulation of attention develops in urban herring gulls. While we did not find a significant difference in approach occurrence and body orientation of adults and immature individuals, herring gulls have an immature period of multiple years (Mullarney et al., 2010) and differences between first winter juveniles and older individuals may be present. More detailed investigations of potential age differences would highlight whether this specific attentional modulation is a learned skill and, if so, when it develops.

Similarly, we may want to ask whether a gull's response would be the same when they pay attention to other animals they have identified as a potential target for kleptoparasitism, or whether these behaviours are human-specific. Previous studies have shown that gulls specifically pick more successful individuals (i.e. those in possession of a larger food item) as food-stealing targets (Busniuk et al., 2020), which may imply that knowledge about their targets allows them to modulate their approach strategy accordingly. As a result, their behavioural response to a non-human individual in possession of food may differ from the responses we present here. Comparisons of how urban gulls attend to food cues from other species and how non-urban gulls react to those from humans will provide more detailed insight into whether urbanisation and frequent contact with people may have caused specific, human-centred behaviours to arise in urban populations.

Lastly, with the ethogram presented here it will be possible to investigate whether attentional cues may be transferred across a group, even when direct visual cues of the food stimulus are lacking. Previous investigations of vigilance in herring gull groups have reported mixed results. Some highlight that vigilance spreads throughout a group, with individuals interrupting their sleep more often to scan their environment if their neighbours are more vigilant (Beauchamp, 2009). Others suggest that groups should follow the ‘many eyes’ hypothesis, according to which individuals decrease their own vigilance when surrounded by highly vigilant neighbours (Roberts, 1996). Using the above-identified attentional markers, we can investigate how vigilance travels through a group of urban gulls, and how the presence of human food cues could affect this.

## MATERIALS AND METHODS

Experiments were conducted by the same experimenter at the Brighton beachfront from March to April 2021 between 7:00 am and 11:00 am on weekdays only, which minimised the chances of pedestrian disturbances. No research was conducted on rainy days to protect the recording equipment. Pilot surveys indicated that gulls preferred to remain in the air on windy days (wind speeds >22.5 km/h) and would not engage with the experimenter during low tide since they were preoccupied with natural foraging in tidal areas. Hence, no experiments were conducted on days on which low tide occurred between 7:00 am and 11:00 am. During all field research, government guidelines around COVID-19 were followed.

Recordings were made with a GoPro Hero 8 or an Honor 20 phone mounted on a LINKCOOL tripod. Video recording was paused or terminated when pedestrians walked into the camera's field of view.

### Ethics

The research was approved by the University of Sussex animal welfare and ethical review body (approval reference ARG/24). It was always ensured that the animals were not disturbed or forced to engage with the experimenter. All gulls had the ability to remove themselves from the situation by either flying or walking away at any time during a trial. The experimenter adhered to the guidelines for the treatment of animals in behavioural research and testing by the Association for the Study of Animal Behaviour (Buchanan et al., 2012).

### Subjects

Single herring gulls (*n*=4) and groups (*n*=9) were approached for observations. For analysis, group sizes were split into individual (*n*=4 birds), small (2–10 birds, *n*=4 groups with a total of 10 birds), medium (11–20 birds, *n*=3 groups with a total of 33 birds), and large (21+ birds, *n*=1 group with a total of 40 birds). The camera was set up about 10 m from the closest individual. We minimised chances of pseudo-replication by alternating trial locations and relying on the large local population size and the species’ fidelity to foraging areas (Clark, 2014; Fuirst et al., 2018; Lato et al., 2021).

Each group was observed for 5 to 20 min, depending on disturbances. Each 5 min block consisted of one of three conditions: a no-food condition (NFC) during which the birds were observed by the experimenter from behind the camera, a food condition (FC) during which the experimenter remained in the same position but retrieved a bag of red Walkers crisps from their backpack, pretending to eat from the bag, and a paper condition (PC) during which the experimenter took a piece of paper from their bag and simulated the rustling noises of a crisps bag by handling the paper along with eating and chewing motions. This would allow for a three-way comparison between a normal control (NFC), a food condition (FC), and a secondary control simulating acoustic and behavioural human food-like cues without presence of a real food item (PC). Other work (Feist et al., 2023) has shown that the colour of a crisp packet does not influence behavioural responses. If groups remained undisturbed and could undergo a complete recording session of 20 min, the following conditions were tested in random sequence: two NFCs, one FC, and one PC, with the two NFCs resulting from the fact that the FC and PC conditions were always separated from each other by an NFC condition.

### Video analysis

Video files were analysed using the Behavioural Observation Research Interactive Software (BORIS) program (Friard and Gamba, 2016). General variables, such as weather condition (sun, cloudy, rain), average temperature in °C, and wind speed in mph during the time of recording, were noted along with low and high tide times and height on survey days. Later analysis showed that weather conditions on recording days only affected gull presence, but not their behaviour. All gulls were given a unique subject identification number and their age was recorded as either immature, adult, or not applicable (NA) if researchers were unsure based on the recorded footage and the age classification provided in Mullarney et al. (2010). Recordings were then analysed until the three selected behaviours of interest, head turns, body orientation relative to the experimenter, and approaches towards them, had been logged for all subjects; videos were watched once per subject, so that on each viewing the experimenter could focus on a single gull. This effectively eliminated the chance of confusing birds within a video. The three selected, distinct behavioural responses were hypothesised to be indicative of attention towards a person or situation (herein food).

### Statistics and modelling

To investigate whether the three selected behaviours were indeed markers of vigilance, statistical analysis focussed on comparing them across conditions. If they were indicative of an increase in attention in a food-related context, it was expected that we would observe more head turns and approaches during FC trials, and that gulls would orient themselves more towards the experimenter. Gulls that participated in the experiment for less than 30 s and those whose age could not be identified from the video footage were excluded from the analysis, resulting in a final *n*=194. Head turns and approaches were counted, and the weighted average angular body position (±0–180°) of test subjects relative to the experimenter was calculated for each individual to obtain one measurement per bird per trial. Furthermore, head turns were divided by the time a gull was in camera view to give ‘head turns per gull minute’, which was used for analysis. All statistical analysis was done in R v.4.1.0 (R Core Team, 2021).

First, a GLMM was constructed to investigate the effects of age (adults versus immature individuals), condition (NFC, FC, and PC), and group size (range: 1–31 individuals; ordinal factor with four levels: individual, small, medium, and large) on head turns. Head turn data was log-transformed to improve the model diagnostics, and trial sequence (1–4) and date were included as random factors. Then, to investigate the effects of age, condition, and group size on the angular body orientation while controlling for trial sequence and date, a second GLMM was built. Variance inflation factor (VIF) analyses were used to investigate correlations among predictors for both models. Lastly, Pearson's Chi-square tests were conducted to confirm whether there was a relationship between condition (NFC, FC, and PC), group size, or age and approach occurrence.
